# “I Was Not Told That I Still Have The Virus”: Perceptions of Utilization of Option B+ Services at a Health Center in Malawi

**DOI:** 10.1177/2325958219870873

**Published:** 2019-09-03

**Authors:** Patience Mulewa, Egrina Satumba, Christopher Mubisi, Joseph Kandiado, Tumaini Malenga, Alinane Linda Nyondo-Mipando

**Affiliations:** 1 Department of Health Systems and Policy, School of Public Health and Family Medicine, College of Medicine, University of Malawi, Blantyre, Malawi

**Keywords:** utilization, Option B+, PMTCT, adherence

## Abstract

Utilization of the prevention of mother-to-child transmission of HIV (PMTCT) services remains a challenge as losses to follow-up are substantial. This study explored factors that influence adherence to maternal antiretroviral (ARV) medications among PMTCT mothers in Malawi. We conducted a descriptive qualitative study from September 2016 to May 2017 using purposive sampling among 16 PMTCT mothers and 4 key informant interviews with health-care workers. Data were audio-recorded and analyzed thematically. The factors that influence adherence to maternal ARV medications include the quality of PMTCT services and social support. Factors that impede adherence include suboptimal counseling women receive on ARV medications, cost of travel, and conflicting advice from religious institutions. Adherence to maternal ARV medications will require the use of existing social support systems in a woman’s life as a platform for delivery of the drugs while also maintaining continued and comprehensive counseling on the benefits of maternal ARV medications.

## Introduction

Prevention of mother-to-child transmission of HIV (PMTCT) refers to a package of interventions for curbing pediatric HIV infection, including maternal HIV testing during antenatal care, maternal and infant use of antiretroviral therapy (ART), infant HIV testing, and safe breastfeeding practices. Malawi, like other countries in Africa, adopted Option B+ as an approach for PMTCT where HIV-infected pregnant and lactating mothers are initiated on triple antiretroviral (ARV) medications for life irrespective of the CD4 count immediately after HIV diagnosis.^1^ Infants on the other hand are initiated on a 6-week program of nevirapine syrup prophylaxis until cessation of breastfeeding and ascertainment of an HIV-negative status.^1^ Malawi championed the implementation of Option B+ approach in July 2011^2^ and later other resource-limited countries adopted the program as well.^3–6^ The decision to offer ARV medications for life to all HIV-infected pregnant and lactating women in Malawi was due to a limited capacity to conduct CD4 count testing for all HIV-infected pregnant women to determine the eligibility for treatment and also to address concerns about the health of women who initiate and later discontinue ARV medications secondary to frequent pregnancies.^7^

The Option B+ approach has increased the number of pregnant and breastfeeding women on ART.^8^ The benefits of the Option B+ approach include improvement in the health status of mother and child and guarantee delivery of an accessible and easy to implement strategy with minimal risk of resistance.^9,10^ Furthermore, the approach addresses stigma and discrimination, the treatment acts as prevention in discordant relationships and offers women active participation in the protection of their baby from HIV.^9,10^ World Health Organization argued that the Option B+ approach reduces stigma through the promotion of breastfeeding even among women who are HIV infected.^9^ The Option B+ approach remains cost-effective for fragile health systems because it simplifies implementation of the PMTCT program and has a simple message to the communities: ART is taken for life.^11^

**What Do We Already Know about This Topic?**

Utilization of PMTCT services is influenced by distance, fear, stigma, social support, and level of knowledge regarding prevention of mother-to-child transmission (PMTCT) services.

**How Does Your Research Contribute to the Field?**

- It addresses the gaps of knowledge on maternal antiretroviral requirements after delivery.
- It highlights the use of “Village Banks” as platforms for promoting adherence to maternal ARV medications.

**What Are Your Research’s Implications toward Theory, Practice, or Policy?**

- Strengthening of available platforms for HIV services such as Village Banks.
- Strengthening HIV counseling in the postpartum period.

Despite the benefits registered with the Option B+ approach in the PMTCT services, there are some operational and clinical challenges.^9^ Utilization of the service by HIV-infected pregnant and lactating women remains below the target. Retention in care is a major challenge in PMTCT services in most countries, including Malawi.^12^ Retention of women in PMTCT programs promotes adherence to ART. Earlier reports indicated that the cumulative losses in sub-Saharan Africa PMTCT programs ranged from 20% to 28% during antenatal care, up to 70% at 4 months postpartum and close to 81% at 6 months after birth.^13^ A recent systematic review reported an improvement in retention with 72.9% at 6 months for studies with less than 12 months of follow-up and 76.4% at 12 months for studies reporting over 12 months of follow-up.^12^ A longer duration of ARV medications is an important factor in achieving viral suppression, and in turn preventing vertical transmission.^14^ Conversely, a loss to follow-up (LTFU) with nonadherence to ARV medications has severe consequences for vertical transmission.^15^ Similarly, in Malawi, utilization of PMTCT services is challenging as evidenced by the LTFU for pregnant and breastfeeding women, which is at 17%. Of the 17%, more losses occurred within 3 months after initiation of ART in facilities with a high patient volume and among women who started ART during pregnancy secondary to Option B+ approach, unlike in the group that initiated ARTs for treatment.^16^ Specifically, at the health center where we conducted the study, from October 2015 to September 2016, the overall LTFU rate among women enrolled in the program was 40%.^17^ Given the prevailing rates of LTFU at the health center, this study assessed the factors that influence adherence to maternal ARV medications among pregnant and lactating women.

## Methods

We conducted a descriptive qualitative study with a phenomenological approach^18^ to understand women’s experiences related to utilization of PMTCT services from September 2016 to May 2017. We assessed health-care worker providers’ and users’ experiences at a health center in Lilongwe, Malawi. A phenomenological design allowed women to share their approach at the health center. We conducted 4 key informant interviews among experts in the provision of PMTCT services at the facility as well as 16 in-depth interviews among HIV-infected pregnant and lactating women attending the PMTCT program to describe women’s experience with PMTCT services and specifically their adherence to maternal ARV medications.

### Study Setting

The health center is an urban health facility owned by the Malawi Government and offers primary-level care. The health center serves an urban, rural, and semirural community with a catchment population of 232 000. The PMTCT services are offered weekdays; HIV Testing and Counseling (HTC) is offered to all pregnant and lactating women, and those diagnosed with HIV are initiated on ARV medications for life on the same day of the diagnosis. During a refill session, health-care providers manually count the pills to ascertain adherence. The health center is also equipped with an electronic medical record system for management of clients on ARV medications which tabulates a simple measure on adherence levels dependent on the number of pills returned against dispensed at the last visit. We selected this health center because of the high rate of LTFU, and the geographical location of the health center provides a variety in terms of socioeconomic status since it serves urban, rural, and semirural communities thereby broadening the scope of the study.

### Selection and Recruitment for HIV-Infected Women

We employed purposive sampling with maximum variation^19^ method to select study participants. The HIV-infected women were included in the study if they were willing to participate in the study. We sought variation in parity, age, distance traveled to the facility, and compliance with the PMTCT schedule in selecting participants for inclusion in the study. A total of 16 HIV-infected pregnant and breastfeeding women enrolled in the PMTCT program were interviewed. Our sample size was adequate according to Guest’s argument that a qualitative researcher would reach almost all codes by the 12th interview thus data saturation.^20^ The study was shared during health education sessions at the facility and study staff approached potential participants as they attended their PMTCT services at the health center. The participants who were adherent to their ARV medications were interviewed within the facility after their appointments. The participants who were nonadherent were identified from PMTCT registers. The Nursing In-Charge of the Maternity section of the facility assisted in identification of nonadherent women following the eligibility criteria. The study staff contacted the potential participants and booked individual appointments with them. None of the identified participants refused participation. All interviews were conducted in a quiet environment within the facility premises and were done in Chichewa.

### Selection and Recruitment of Key Informants

We drew a purposive sample^21^ of health-care workers who worked in different sections of PMTCT services at the health center, such as HIV testing and counseling, administration of ARV medications to clients, mentoring of HIV-infected pregnant and breastfeeding women, and follow-up of women in the program. The Nursing Sister In-charge of the maternity section assisted the study team in selecting eligible health-care workers for the study. All interviews with health-care workers were done in their respective offices at the facility. The sample size for key informants was small because being a health center, the number of health-care workers is very small hence a limited number of personnel to draw from. We included key informants to gain their perceptions on adherence to maternal ARV medications. All health-care workers who were approached accepted to participate in the study.

### Data Collection

Interview guides were developed in English, translated into Chichewa, and were piloted at the same center prior to the study rollout. All interviews were face-to-face and audio-recorded with consent from the participants using a digital voice recorder and lasted about 30 to 45 minutes. The research team conducted all the interviews in Chichewa. All researchers are health-care workers but were not employed at the study site and had no prior relationship with study participants. The researchers introduced themselves as health management students when they solicited consent for participation from the study participants. Two of the researchers were female while the other 2 were male. Pregnant and lactating women were asked about motivating factors for them to utilize the PMTCT services and why they remain adherent or nonadherent to maternal ARV medications. The key informants were asked about perceived factors for engaging and disengaging with services including adherence and nonadherence to maternal ARV medications. The questions are as follows:

1. Explain to me in detail the factors that promote adherence to ARV medications?
2. Explain to me in detail the factors that challenge adherence to ARV medications?

Under each question we probed on individual, health system and community level factors that may influence adherence to maternal ARV medications. Data from audio-recorders were transferred into computers that were password protected. We triangulated^22^ the data collected from both service users and providers to increase the validity of our results. We also employed member checking^23^ with all participants by summarizing findings from each participant after an interview on the same day, for verification of key points to ensure the credibility of our findings.

### Data Analysis

We analyzed the data manually using thematic analysis as suggested by Braun and Clarke.^24^ Audio-recorded interviews were transcribed and translated into English by the research team as part of data familiarization. Four members of the research team coded the data inductively from the data and deductively from the objectives. Further familiarization of the data set was achieved by reading and rereading the transcripts to gain a full picture of the data. Four members of the research team individually coded 1 transcript and compared the codes for any similarities and differences in coding. The coding team discussed the areas where they coded differently to a consensus on the most appropriate codes. Each of the 4 researchers coded the initial transcripts then independently coded the rest of the transcripts following the coding guide and they checked one another’s coded transcripts for completeness and accuracy. Codes that were similar were grouped together under an overarching theme. Thereafter, we defined and named the themes after an iterative process of comparing the codes and the themes to achieve the best fit.

### Ethical Considerations

We obtained ethical clearance from College of Medicine Research Ethics Committee (COMREC-Number BScHM/02/17/17) prior to implementation of the study. We sought permission for data collection from the officer-in-charge of the health center. The research team ensured that privacy, confidentiality, and rights of participants were observed during and after the study. A written informed consent for participation in the study was obtained from each study participant. Illiterate participants thumb printed the form after it was read to them in the presence of an impartial witness. All study deliberations were done in Chichewa for the HIV-infected women and in some cases, we included English for the health-care workers. We used pseudo names to identify the study participants. We also completed the Consolidated Criteria for Reporting Qualitative Studies Checklist for the study (Supplemental Appendix A) to remain compliant with reporting of qualitative studies.

## Results

### Characteristics of HIV-Infected Women

A total of 16 women were interviewed. Among the women interviewed, 7 were pregnant at the time of interview. The age of the women ranged from 18 to 49 years, 12 women were married, 9 were literate, 14 were unemployed, 1 was self-employed, and 1 was employed. Two were primigravidae and 11 had 1 to 3 children (Table 1).

**Table 1.** Characteristics of the HIV-Infected Women.

Variable	Adherent	Nonadherent
Pregnant	4	3
Lactating	4	5
Age		
18-29	4	4
30-39	4	2
40-49	0	1
Unknown	0	1
Marital status		
Married	8	4
Single	0	2
Other	0	2
Literacy		
Yes	5	4
No	3	4
Number of children		
0	1	1
1-3	5	6
4-6	2	1

### Characteristics of the Key Informants

The participants included the following: 2 ART providers, 1 mother-2-mother mentor, and 1 HTC counselor/community worker. All health-care workers were involved with the provision of PMTCT services at different levels of care.

### Lived Experiences of Women with PMTCT Services

The factors that influence compliance to maternal ARV medications and continued utilization of PMTCT services were categorized under 2 overarching themes and classified under enablers and barriers. The 2 themes are quality of PMTCT services and social support (Table 2).

**Table 2.** Factors That Influence Utilization of PMTCT Services.

Theme	Enablers	Barriers
Quality of PMTCT services	Maternal and infant health benefits	Suboptimal quality of PMTCT counseling
	Nutritional benefits	Geographical and financial accessibility
	Provider’s attitude	
	Adherence as a measure of preserving privacy	
Social support	Family and spousal support	Stigma
	Support groups	Nondisclosure of an HIV-infected status to a partner
		Unsupportive spouse
		Conflicting religious advice

Abbreviations: PMTCT, mother-to-child transmission of HIV.

## Factors That Influence Adherence to Maternal ARV Medications

### Theme 1: Quality of PMTCT Services

#### Quality of PMTCT services: barriers to adherence to maternal ARV medications

##### Suboptimal quality of PMTCT counseling

The adequacy of counseling received by a woman influenced her utilization of PMTCT services including adherence to maternal ARV medications. This level of counseling resulted in a woman having limited knowledge of the benefits of continued use of PMTCT services. One respondent said,

I wasn’t told that I still have the virus or not. They just said that I should be taking the drugs to prevent transmission to the baby. (Lactating woman, nonadherent)

A key informant confirmed that in other instances women are not adequately counseled.

It’s because they did not receive enough counseling when they were found HIV-positive and during initiation of treatment and did not understand this made them to have problems in attending antenatal clinic.(KII3)

The inadequate counseling received limits the information women may have on the side effects of ARV medications and their management which inevitably leads to nonadherence to maternal ARV medications. A woman who was nonadherent to their ARV medications stated:

I was having epileptic seizures during the night since I started taking ARVs…which led to stopping them (ARVs). (Lactating woman nonadherent)

In the presence of adequate information, a woman adheres to her ARV medications irrespective of the presence of side effects. A woman who was adherent to her ARV medications narrated her experience with side effects from ARV medications and the advice she received from a health-care worker as follows:

For the heart palpitations and dizziness, I have been having them when I was one month pregnant, but the heart burn after starting medication and the doctor said that they are minor side effects which are common when you have just started medication but stops later. (Pregnant woman, adherent)

Additionally, inadequate counseling coupled with limited knowledge perpetuates nonadherence:

They advise me to be compliant to medication only that it’s my negligence, even my husband reminds me. (Lactating woman, nonadherent)

For some women it is due to negligence, they can collect the medication and just leave them home, some can only come once and never come back. (KII 2)

#### Quality of PMTCT services: enablers to adherence to maternal ARV medications

##### Maternal and infant health benefits

The importance of taking ARV medications for their own health and protecting the baby was the major driving force for most women to adhere to ARV medications and continually engage with the PMTCT services. The goal of having an HIV-free child motivated women to keep on using the services. The perceived benefits of an HIV-free child were mostly reported by lactating women.

The baby I am expecting should be protected from the virus…even myself to stay healthy…to protect the unborn child from the virus and also to keep me healthy so that l can live long. (Lactating woman, adherent)

I want to prolong my life, raise my children, leaving them whilst they are old enough. (Lactating woman, adherent)

A child’s negative HIV result after 2 years of attendance at a PMTCT services encourages women to continuously use the services. A key informant reported as follows:

We have a lot of evidence, those children when tested and are (HIV) negative after two years, this helps those women who want to be pregnant again or those that are already pregnant to make sure that they protect the baby in order to be born HIV-negative. (KII 4)

##### Nutritional benefits

The material and health benefits that women get from the services motivate them to keep using the services. The nutrition support the women receive in form of Vitameal flour promotes service utilization.

Every pregnant woman who comes to this facility is attached to a person who follows them at their homes so that they should start receiving flour for porridge. This is because the drugs make us to be very hungry so we are supposed to take porridge early in the morning. (Lactating woman, adherent)

We have partners who provide flour for porridge, as for us we don’t provide anything; we used to conduct support groups but stopped due to other circumstances. Provision of the flour encourages these women. (KII2)

##### Positive attitude of health providers

Good interaction with clients at the ART clinic also served as a motivating factor to utilization of services at each point in the PMTCT care cascade. Participants expressed satisfaction with the care, reception, and assistance with their complaints at the facility.

I know many people here including the doctors who advise me well on how to live positive and take the drugs. (Lactating woman, adherent)

We are being assisted because when we come for drug collection, we are also able to give complaints to the doctors…during the first visit, I was told that they are minor side effects. (Pregnant woman, adherent)

We have proper procedures of keeping them waiting, good waiting place, and are welcomed by health-care workers. There is good interaction with them; this makes a person to return to the hospital. (KII4)

##### Adherence as a measure of preserving privacy

Participants adhered to their ARV medications and collection schedule to avoid health-care workers from following them to their homes, which may compromise privacy. One health worker explained their tactics as follows:

The main reason for compliance (ART services) is because of our skills and we advise them (patients) to make sure that they meet us each and every time they come for their visits and if they fail to do so, we have the right to follow them to their homes, and many people know us that we are from the hospital, so if we follow them to their homes they will be embarrassed. (KII2)

### Theme 2: Social Support

#### Social support: barriers to adherence to maternal ARV medications

##### Stigma

Participants shared how stigma affected their utilization of PMTCT services. Stigma was displayed in the form of mockery, gossip, and ill-treatment. The following excerpts illustrate this.

My friends laugh at me whenever I meet them. I avoid meeting them and mostly stay at home. (Pregnant woman, nonadherent)

He (participant’s husband) is fond of reminding me about the past and uses abusive language…He says I have to tell the one who gave me the disease (HIV) so that he can take care of me whether it is money for buying food for the child but the one who infected me passed away. (Lactating woman, nonadherent)

##### Nondisclosure of an HIV-infected status to a partner

The health-care workers stated that nondisclosure of HIV-positive test results to partners perpetuated nonutilization of services at each point in the PMTCT care cascade and nonadherence to maternal ARV medications. The reasons for nondisclosure included fear of being divorced and abused. The women in the study reported to have disclosed to their partners and other relevant people in their lives.

Most problems as I said are due to non-disclosure of status to spouses, which is leading to non-compliance. (KII2)

Their (women’s) hindrance to come here (health centre) mostly is due to reasons from their homes. Sometimes it’s because they didn’t disclose their status so they don’t see any need for coming here. (KII 4)

##### Unsupportive spouse

Absence of support from partners, relatives, and communities contributed to nonadherence to maternal ARV medications. One woman stated:

When it’s my appointment date, he does not allow me to go and access the drugs and says if you go using your own power do not attempt to come again at this house because of this. I do not go but this is not because of my own will but is because of the problems that I meet within the house. (Lactating woman, nonadherent)

A health-care worker corroborated the assertion by women and reiterated that women without support rarely continued attendance to PMTCT services.

Some come without their spouses and this makes it very difficult for the mothers to disclose their status once found positive. (KII3)

##### Conflicting religious advice

Other religious leaders discourage women from taking ARV medications on the basis that prayers can heal. One woman explained this as follows:

My pastor prayed for me and declared that am healed from my sickness and advised me to stop the drug and have hope that am healed. (Lactating woman, nonadherent)

Some stop their ARVS due to religious beliefs, after being prayed for by pastors who tell them that they are healed and ought to have hope. This led to more women stopping the treatment. (KII 4)

#### Social support: enablers to adherence to maternal ARV medications

##### Family and spousal support

The various forms of social support systems around a woman motivate her to continuously take her ARV medications. This support could be from a partner, relations, and her community. Social support from partners and relatives is key in facilitating utilization of services at each point in the PMTCT care cascade.

My mum encourages me, she tells me that it’s not the end of life but the beginning of a new one, this facilitates my daily uptake of the drugs…am not stressed, even my husband doesn’t know. (Pregnant woman, adherent)

I thank God almighty for having a lovely husband who mostly encourages me to continue taking these drugs. (Lactating woman, adherent)

Additionally, the financial support that a woman receives from her family motivates her to continue using PMTCT services.

Honestly we are trying hard at home…my husband works hard in sourcing food for me for example eggs, fish sometimes meat. (Pregnant woman, adherent)

Health-care workers encourage the presence of a guardian (this is a person selected by the client who knows about the client’s status and regimen) who is counseled alongside the woman on her ARV medications with a goal of widening the support system for the woman. Guardians are key in reminding a woman to take her ARV medications and compliance to her clinical appointments.

We (health care workers) make sure that there is somebody from where they (women) are coming from, whom they have disclosed their status; and this person must be either a relative or husband, we encourage the husband that whenever she has forgotten taking the drugs, he has to remind her taking the drugs. (KII1)

##### Support groups

Community support through village social groups is another forum that motivates utilization of PMTCT services including adherence to maternal ARV medications. During these gatherings, HIV-infected women share information among themselves and encourage one another to remain adherent to ARV medications.

We have created a village group of women of my status (HIV infected) where so many issues are discussed and solutions are made.(Lactating woman, adherent)

Volunteers come from the villages where our clients stay and have competence in teaching, whether to positive or negative, they reach every where even to village groups gatherings such as Bank mukhonde (Village Banking Groups are informal and community based banking institutions that are managed and created by community members), in churches and any community gatherings where necessary in the villages. (KII 4)

## Discussion

The factors that affected utilization of PMTCT services and adherence to maternal ARV medications included quality of PMTCT services and social support. The factors that contributed to nonadherence to maternal ARV medications were suboptimal quality of PMTCT counseling, stigma, unsupportive spouse, nondisclosure of an HIV-infected status to a partner, conflicting religious advice, and geographical and financial accessibility. The factors that promoted adherence to maternal ARV medications were maternal and infant health benefits of ARV medications; nutritional supplements received from the service; the presence of family, spousal and guardian support; support groups; and adherence as a measure of preserving privacy. The existing social support around a woman formed the bedrock for the decisions made on adherence to maternal ARV medications.

### Barriers to Adherence to Maternal ARV Medications

The lack of knowledge on the importance of taking ARV medications influenced nonadherence to the PMTCT services. This finding builds on what was reported in Lilongwe that adherence to the Option B+ approach was influenced by knowledge of perceived importance and consequences for not adhering to ART^25^ and that lack of knowledge negatively impacts the uptake of PMTCT services.^26,27^ Similarly, among postpartum women in PMTCT services, ignorance of the whole program contributed to lack of utilization.^28^ Some women in our study stopped their ARV medications postnatally, which remains consistent with findings from a South African study that stated that some women were only interested in having an HIV-free child after which they would stop attending to PMTCT services postnatally.^28^ This practice may illustrate inadequate knowledge on the transition paths of HIV postnatally and also supports what Tweya et al^29^ revealed earlier on the lack of understanding on the initial counseling at point of ART initiation. We argue that targeted postnatal counseling be implemented to ensure that women are supported with information on the PMTCT services and benefits of ARV medications at each stage of the maternity cycle.^29,30^ Adequate knowledge of PMTCT^25,31^ and the belief in the effectiveness of the ARV medications^32^ facilitates utilization of PMTCT services.

Similar to a study in Papua New Guinea, our study revealed that that there were some women who discontinued their ARV medications despite being knowledgeable of PMTCT services and having support from family and relatives and this result contradicts earlier studies reported in Malawi and Uganda.^32,33^ Previous studies have asserted that in some instances women who discontinued ARV medications felt pressure to start on ARV medications while pregnant on the same day of diagnosis.^34^ Furthermore, during the postpartum period some women stopped taking ARV medications to avert inevitable disclosure that comes along following delivery since a woman can no longer disguise ARV medications as prescribed prenatal medicines.^28^ Additionally, it has been documented that asymptomatic women are less motivated to engage with PMTCT services nor adhere to their ARV medications.^32^ Although women cited side effects as a factor for discontinuing their ARV medications in this study and other previous studies,^35,36^ the ARV medications currently in use have fewer side effects when compared to the old regimen that phased out^37^ and have potential of limiting nonadherence secondary to side effects. Comprehensive education on the expected side-effects of ARV medications may lessen nonadherence and disengagement from services. The education could be summarized in an information sheet that could be shared with the women or in an audio-visual format that could be displayed at the facility for women to watch as they access services.

The fear of losing a marriage which is intertwined with social support and lack of disclosure in our study remains consistent with a study in Lilongwe where women refrained from PMTCT services because they feared the indirect disclosure of an HIV-infected status, which could result into abandonment and divorce.^30,38^ A study conducted in 4 African countries reported that women felt rejected and some were divorced after disclosure.^33^ To avoid dissolution of marriage, some women delayed initiation of ARV medications until they had discussed their involvement with their male partners,^37^ which underscores the importance of male involvement in PMTCT for adherence purposes.^39^ We argue for strengthening of facilitated mutual disclosure patterns^35,40^ to achieve optimal utilization of services. Earlier studies reiterated that nondisclosure of an HIV-infected status secondary to fear of stigma made it difficult for women to collect their ARV medications from a clinic and promoted covert uptake of ARV medications.^27,28,32,41,42^ As alluded by earlier studies,^4,43^ some religions discourage the use of ARV medications on the premise that one is healed which highlights the relevance of engaging faith and religious sectors in HIV services.

Our results show that women attributed geographical distance and financial constraints as contributing to nonadherence to ARV medications. The findings build upon other studies conducted in Malawi^29,35^ and studies done elsewhere.^4,27,28,42,43^ Another study in Malawi highlighted that LTFU in Lilongwe was more in the rural areas and among pregnant and breastfeeding women.^29^ Our study was conducted in a facility that serves both rural and urban clients which may explain the observed lack of utilization. Notably, during our study there were some women who covered the same distance to the health facility but still were adherent to their ARV medications which suggests that the variations were contextualized at an individual level.^44^ Geographical and financial constraints may be curbed by introducing mobile ART clinics to limit the distance women have to cover. A study among the general population accessing ARV medications noted that HIV-infected people travel longer distances when compared with those who are HIV uninfected to access services.^45^

### Enablers to Adherence to Maternal ARVs

Our findings on support groups through social groups like “Village Banks” that motivate women to use PMTCT services are unique. Village Banks are financial institutions that are formed and managed by community members to promote access to banking benefits locally and do not receive any financial support from any institution.^46^ Village Banks offer a self-help service as money lending and savings institutions^46^ and build on behavioral economics^46^ aspects of social reference and temporal salience. Social reference emphasizes on the support from others with a similar condition to support those who are newly diagnosed to use the intended services and temporal salience asserts that the presence of people with similar situations around a person offers them the reference point one may need.^47^ In previous studies, the support groups also proved to be one of the facilitators to compliance.^48^ Additionally, the support from partners and relatives expressed in this study is related to studies that have highlighted on male involvement and its ability to enhance PMTCT compliance across the different steps.^40,49^ Other modes of support could be in the form of text messages^49^ which was not expressed in our study, partially because mobile health is not widely used in health services in Malawi despite most people owning mobile phones. Mobile texts to women reminding them of appointment dates led to higher rates of infant HIV testing in a postpartum PMTCT clinic in Kenya.^50^

A positive relationship between health-care providers and clients facilitates utilization of PMTCT services and adherence to maternal ARV medications.^27^ Conversely, studies have reported that complexity of patient–provider relationship in which negative attitudes or disrespectful providers were displayed was a barrier to care.^42,51^ A previous study suggested that an improved provider–client communication through education and good health-care worker communication skills could enhance adherence to ARV medications.^52^

In our study, HIV-infected pregnant and breastfeeding mothers enrolled in a PMTCT program were encouraged and motivated to come and collect their ARV medications because of the provision of Vitameal flour each time they come to access the treatment. The study also revealed that women were drug compliant because they were aware of the benefits of ARV medications on their health. Similarly, it has been previously reported that benefits related to the PMTCT program encourage women to continue with ARV medications.^41^ Other studies have also shown that the desire to have an HIV-negative baby has been a motivator for attending PMTCT programs.^37^ Furthermore, a recent study in Malawi showed that the desire of women to keep themselves and their child healthy motivated them to remain adherent to ARV medications.^47^ As part of the Option B+ approach, it will be important to continuously link the infant’s and mother’s health in a way that motivates women to utilize the services both during pregnancy and beyond.^47^ Although women in our study stated the desire to have an HIV-free child as a motivating factor to continuously use the services as has been reported previously,^34^ a South African study showed that women would stop attending to PMTCT services after the birth of their child as they were busy with caring for the baby.^28^ Furthermore, nonadherence to the drug regimen despite desiring an HIV-uninfected child could be due to the instant initiation of a lifelong drug regimen,^37^ which diminishes the amount of time for a woman to process and accept the implications of her status.^34^

Strategies that may be explored would be community-based ART clinics^53^ that build upon social groups already in existence in the community. Community health-care workers^34^ could serve as the link between women and health facility in strengthening information shared on PMTCT services.

### Study Strengths and Limitations

The strength of our study is that it presents the lived experiences of HIV-infected pregnant and lactating women who have remained adherent or nonadherent to the PMTCT services and perceptions of service providers. However, this study was done at 1 health center on a small scale using a sampling technique that does not allow for generalization, hence the results may be limited to this health facility. We also depended on perceptions and personal reports which yield different results as opposed to observing behaviors. Our findings build on the existing literature and specify other means within a woman’s context that promotes utilization of PMTCT services that could be built upon to improve compliance to services. Although we have recommended Village Banks as a platform for optimizing adherence to maternal ARVmedications, we recommend that future research focus on feasibility and acceptability of Village Banks as a platform for improving adherence to maternal ARV medications.

## Conclusion

Continued utilization of PMTCT services and adherence to maternal ARV medications is influenced by a woman’s perceptions of ARV medications, forms of support available to the woman, and accessibility of the services. Attention to patient-centered care and targeted health facility interventions may improve utilization of PMTCT services. Additionally, as HIV and AIDS become a chronic condition, there is a need to capitalize on the community-based support groups that may support the health system to optimize adherence to maternal ARV medications.

## Supplemental Material

Supplemental Material, COREQ_Final_June_2019 - “I Was Not Told That I Still Have The Virus”: Perceptions of Utilization of Option B+ Services at a Health Center in Malawi Click here for additional data file. Supplemental Material, COREQ_Final_June_2019 for “I Was Not Told That I Still Have The Virus”: Perceptions of Utilization of Option B+ Services at a Health Center in Malawi by Patience Mulewa, Egrina Satumba, Christopher Mubisi, Joseph Kandiado, Tumaini Malenga and Alinane Linda Nyondo-Mipando in Journal of the International Association of Providers of AIDS Care (JIAPAC)
