# Supplementary material for: “I Was Not Told That I Still Have The Virus”: Perceptions of Utilization of Option B+ Services at a Health Center in Malawi
Source: J Int Assoc Provid AIDS Care. 2019 Sep 3;18:2325958219870873. doi: 10.1177/2325958219870873 (PMC6900569; doi:10.1177/2325958219870873)
Supplement: Supplemental Material, COREQ_Final_June_2019 - “I Was Not Told That I Still Have The Virus”: Perceptions of Utilization of Option B+ Services at a Health Center in Malawi [file COREQ_Final_June_2019.docx]

**Consolidated criteria for reporting qualitative studies (COREQ): 32-item checklist**

| **No. Item** | **Guide questions/description** | **Reported on Page #** |
| --- | --- | --- |
| **Domain 1: Research team and reﬂexivity** |  |  |
| *Personal Characteristics* |  |  |
| 1. Inter viewer/facilitator | Which author/s conducted the inter view or focus group? | Results: PM, ES, CM and JK conducted the interviews (currently blinded until after peer review) |
| 2. Credentials | What were the researcher’s credentials? E.g. PhD, MD | Methods: PM , ES, CM and JK are Health Managers while TM is a social scientist and ALNM is a Health System Specialist. t(currently blinded until after review) |
| 3. Occupation | What was their occupation at the time of the study? | Methods  PM,ES, CM and JK were Health Management students while TM is a PhD student and ALNM is a Health Systems Specialist supervising the group of Health management students (Currently hidden until after peer review) |
| 4. Gender | Was the researcher male or female? | Two were female and two were males, page 8. |
| 5. Experience and training | What experience or training did the researcher have? | Methods- The four data collectors were health management students. Page 7-8 |
| *Relationship with participants* |  |  |
| 6. Relationship established | Was a relationship established prior to study commencement? | All researchers are health care workers but were not employed at the site where the study was conducted and had no prior relationship with study participants. They introduced themselves at the time of obtaining consent from the potential participants. page 7-8 |
| 7. Participant knowledge of the interviewer | What did the participants know about the researcher? e.g. personal goals, reasons for doing the research | All researchers are health care workers but were not employed at the site where the study was conducted and had no prior relationship with study participants. They introduced themselves at the time of obtaining consent from the potential participants. page 7-8 |
| 8. Interviewer characteristics | What characteristics were reported about the inter viewer/facilitator? e.g. Bias, assumptions, reasons and interests in the research topic | They introduced themselves as students at the time of obtaining consent from the potential participants. page 8 |
| **Domain 2: study design** |  |  |
| *Theoretical framework* |  |  |
| 9. Methodological orientation and Theory | What methodological orientation was stated to underpin the study? e.g. grounded theory, discourse analysis, ethnography, phenomenology, content analysis | Methods- a descriptive qualitative study with a phenomenological approach, page 5 |
| *Participant selection* |  |  |
| 10. Sampling | How were participants selected? e.g. purposive, convenience, consecutive, snowball | Methods- Page 6-7, we drew a purposive sample. |
| 11. Method of approach | How were participants approached? e.g. face-to-face, telephone, mail, email | Methods- Page 7, under data collection, all interviews were face to face. |
| 12. Sample size | How many participants were in the study? | Results- there were 24 participants in the study. On page 10 under characteristics of participants. |
| 13. Non-participation | How many people refused to participate or dropped out? Reasons? | Methods  None of the identified participants refused participation. Page 7 |
| *Setting* |  |  |
| 14. Setting of data collection | Where was the data collected? e.g. home, clinic, workplace | Methods: The data was collected at the clinic after attendance to clinic duties or appointments. This is on page 8-9 |
| 15. Presence of non-participants | Was anyone else present besides the participants and researchers? | Methods  No |
| 16. Description of sample | What are the important characteristics of the sample? e.g. demographic data, date | Results  Under demographic characteristics of participants on page 10 |
| *Data collection* |  |  |
| 17. Interview guide | Were questions, prompts, guides provided by the authors? Was it pilot tested? | Methods- on page 7 under data collection, there is information on piloting and the questions asked. |
| 18. Repeat interviews | Were repeat inter views carried out? If yes, how many? | N/A |
| 19. Audio/visual recording | Did the research use audio or visual recording to collect the data? | Methods- page 7-8 specifies that we audio recorded the interviews. |
| 20. Field notes | Were ﬁeld notes made during and/or after the inter view or focus group? | Methods- Page 8 specifies that field notes were taken |
| 21. Duration | What was the duration of the inter views or focus group? | Methods-Page 7 specifies that it was 30-45 minutes. |
| 22. Data saturation | Was data saturation discussed? | Methods- Page 8 discusses data saturation |
| 23. Transcripts returned | Were transcripts returned to participants for comment and/or correction? | N/A |
| **Domain 3: analysis and ﬁndings** |  |  |
| *Data analysis* |  |  |
| 24. Number of data coders | How many data coders coded the data? | Methods- Under Data Analysis on page 8-9, Four members of the research team coded the data inductively from the data and deductively from the objectives. Further familiarization of the dataset was achieved by reading and rereading the transcripts, to gain a full picture of the data. Four members of the research team individually coded one transcript and compared the codes for any similarities and differences in coding. |
| 25. Description of the coding tree | Did authors provide a description of the coding tree? | N/A |
| 26. Derivation of themes | Were themes identiﬁed in advance or derived from the data? | Methods- Themes were derived from the data as in page 9 |
| 27. Software | What software, if applicable, was used to manage the data? | Page 8 states that we manually managed and analyzed the data |
| 28. Participant checking | Did participants provide feedback on the ﬁndings? | Strengths and limitations- We employed member checking by summarizing the key findings of each interview after each interview as on page 9. |
| *Reporting* |  |  |
| 29. Quotations presented | Were participant quotations presented to illustrate the themes/ﬁndings? Was each quotation identiﬁed? e.g. participant number | Results- All our results have quotes that substantiate them. See pages 10-21. |
| 30. Data and ﬁndings consistent | Was there consistency between the data presented and the ﬁndings? | Relationship to existing knowledge- Our discussion pages 21-26 is situated in existing literature. |
| 31. Clarity of major themes | Were major themes clearly presented in the ﬁndings? | Results- we summarized into major theses as in pages 10-21 |
| 32. Clarity of minor themes | Is there a description of diverse cases or discussion of minor themes? | Discussion- Minor themes are presented under each theme in pages 10-21. The minor themes are discussed in the discussion as well. |
